# Parental supervision and sexual behavior among Brazilian adolescents

**DOI:** 10.1590/1980-549720230013.supl.1

**Published:** 2023-04-21

**Authors:** Gleice Barbosa Reis, Marco Aurélio de Sousa, Gisele Nepomuceno de Andrade, Deborah Carvalho Malta, Ísis Eloah Machado, Mariana Santos Felisbino-Mendes

**Affiliations:** I Universidade Federal de Minas Gerais, Nursing School – Belo Horizonte (MG), Brazil.; II Universidade Federal de Minas Gerais, Nursing School, Department of Maternal-Child Nursing and Public Health, Postgraduate Program in Nursing – Belo Horizonte (MG), Brazil.; III Universidade Federal de Ouro Preto, Department of Family, Mental and Collective Health, Postgraduate Program in Health and Nutrition – Ouro Preto (MG), Brazil.

**Keywords:** Unsafe sex, Family development planning, Adolescent, Parent-child relations, Sexual and reproductive health, Gender inequality

## Abstract

**Objective:**

to evaluate the association between parental supervision and sexual behaviors among Brazilian adolescents.

**Methods:**

Cross-sectional study with data from 102,072 adolescents who responded to the National Adolescent School-based Health Survey. We estimated the prevalence of sexual behaviors (initiation, use of condoms, contraception, and number of partners). Parental supervision was evaluated using a score considering five indicators. We calculated prevalence ratios (PR) adjusted by age and sex in order to estimate the association between parental supervision score and sexual behaviors of adolescents.

**Results:**

Prevalence of risky sexual behavior for adolescents with minimum and maximum parental supervision were: sexual initiation (min.: 58.0%; max.: 20.1%), condom use in the last sexual intercourse (min.: 50.9%; max.: 80.2%), use of contraceptives (min.: 40.8; max.: 49.1%), and mean number of partners (min.: 3.25; max.: 2.88). Parental supervision was greater among girls. Those with higher supervision scores had higher prevalence of condom use in the first and last sexual intercourse and of contraceptive methods, and a smaller mean number of partners, even after adjustments for sex and age.

**Conclusion:**

The greater the parental supervision, the better the sexual behavior for both sexes, although supervision seems to occur differently between girls and boys. These findings point to the role of the family in providing adolescents with monitoring, along with dialogue and affection, conditions that encourage healthy and risk-free sexual behavior.

## INTRODUCTION

Adolescence is marked by bodily, cognitive, emotional and social transformations that motivate changes in behavior patterns and demand greater family attention^1,2^. The possibilities of learning, discoveries and experiments in this age group are known to lead to situations of risk and vulnerability, especially when related to sexuality^3^. Unsafe sexual practices occur mainly due to taboos in the discovery and exercise of sexuality, lack of information and lack of communication with family members^4^.

With regard to family, parental supervision—a type of indirect monitoring of the behavior of adolescents^5^ that includes the application of rules and parental knowledge about the location and activities of their children, as well as dialogue, affection, among other factors—, can contribute to modify the sexual behavior of adolescents^2,6^. Previous studies have shown that higher levels of parental monitoring are related to a greater probability of delaying sexual initiation^6^ and of using condoms and other contraceptive methods^2,7,8^, contributing to the adoption of healthy sexual practices and reproductive health by adolescents. The protective effect of parental supervision on other health outcomes was also seen^2,6,7,9^.

International,^2,6,10^ national^11,12,13^, regional^1,4,14,15^ and local studies, such as in a rural population^16^, have increasingly investigated the sexual behavior of adolescents, including their relationship with parental supervision. However, studies that consider gender differences are less frequent and address few parental supervision indicators^8^, generally related to knowledge about where the teenager is when not at school, which refers to supervision regarding limits and responsibilities. However, issues that refer to dialogue and affection should also be considered^10^. A review study showed differences in supervision depending on gender, with boys receiving more supervision related to risk sexual behaviors while girls receiving more emotional support^10^.

In this sense, this study aimed to evaluate the association between parental supervision and sexual behaviors adopted by Brazilian adolescents of both sexes. It is hypothesized that adolescents with greater parental supervision present lower-risk sexual behaviors.

## METHODS

This is a cross-sectional study that analyzed data from Sample 1 of the National Adolescent School-based Health Survey (PeNSE) carried out in 2015, comprising 102,072 adolescents enrolled in and regularly attending public and private schools located in urban and rural areas throughout the national territory in 2015^17^. The survey was carried out by the Ministry of Health (MS) in partnership with the Brazilian Institute of Geography and Statistics (IBGE) and the Ministry of Education (MEC).

### Sample design and data collection

Sample 1 of PeNSE 2015 was planned to estimate population parameters in Brazil's Major Regions, Federative Units and municipalities of capitals^17^. For the sampling plan, 53 geographic strata were defined. Each of the 26 state capitals, plus the Federal District, was defined as a geographic stratum, and the other municipalities were grouped into another 26 strata representing each Brazilian state, excluding the capitals^17^. In the 53 strata formed, a sample of schools was selected based on data from the 2013 School Census^17^. To ensure the presence of public (federal, state or municipal) and private schools in an approximate proportion to their existence in the selected register, allocation strata were formed by crossing geographic strata with the network of schools (public or private) and their size, measured by the number of 9th grade classes^17^.

In each school in the sample, the 9th grade classes were selected by lot, and the respective students present on the day of data collection were invited to respond to the research questionnaire using a smartphone. The questionnaire was divided into thematic modules addressing various risk and protective factors, including questions on parental supervision received and on sexual and reproductive health^17^. Further information on the PeNSE sampling plan and data collection methodology can be found at: https://www.ibge.gov.br/estatisticas/sociais/educacao/9134-pesquisa-nacional-de-saude-do-school.html?=&t=what-is

### Variables

Parental supervision was the variable of interest in this study. The five questions used to proxy parental supervision are shown in Chart 1. In addition to assessing each variable, the responses of the five variables were added up, generating a score that ranged from 0 to 5. Thus, adolescents whose score was equal to 0 (“yes” for the variable that analyzes missing classes without parental permission and “no” for the other variables) were considered as little or no parental supervision, and those whose score was 5 (“no” for the variable that analyzes missing classes without parental permission and “yes” for the other variables) were classified as “under intense supervision”.

**Chart 1:** Parental supervision and categorization of variables.

Variables	Question	Categorization	Score
Missing class without parental permission	IN THE PAST 30 DAYS, on how many days did you miss classes or school without permission from your parents or guardians?	Yes — 3 days or more in the last 30 days	0
		No — 2 days or less in the last 30 days	1
Parents aware of free-time activities	IN THE PAST 30 DAYS, how often did your parent or guardian really know what you were doing in your free time?	Yes — most of the time or always	1
		No — never, rarely, or sometimes	0
Parents check that homework has been done	IN THE PAST 30 DAYS, how often did your parents or guardians check that your homework was done?	Yes — most of the time or always	1
		No — never, rarely, or sometimes	0
Parents understand their problems and concerns	IN THE PAST 30 DAYS, how often did your parents or guardians understand your problems and concerns?	Yes — most of the time or always	1
		No — never, rarely, or sometimes	0
Parents present at meals often	Do you usually have lunch or dinner with your parents or guardian?	Yes — 5 or more days a week	1
		No — 4 or fewer days a week	0

Sexual behavior among adolescents was assessed using the following variables: sexual intercourse at least once in life (initiation); use of condom in first sexual intercourse, use of condom in the last sexual intercourse, use of a method to avoid pregnancy in the last intercourse and number of sexual partners in life.

The other covariates assessed were sex (male, female) and age group (11–13, 14–16, 17–19).

### Data analysis

The prevalence of sexual behavior indicators among Brazilian adolescents was estimated according to parental supervision received. Then, the prevalence of parental supervision variables according to adolescents’ gender was estimated. The average number of sexual partners reported by adolescents was also calculated, according to parental supervision, for both genders. Statistical differences were measured using Pearson's χ2 test, with a significance level of 5%, and 95% confidence intervals (95%CI).

Finally, crude and age-adjusted prevalence ratios (PR) of adolescents’ sexual behaviors were estimated according to parental supervision scores using Poisson regression, considering the magnitude of the prevalence of sexual behaviors^18^. For the variable number of sexual partners, crude and adjusted linear regressions were performed to estimate differences in means. For all analyses, respective 95%CI were estimated.

Adolescents who stated that they had not initiated their sexual lives (n=72,989) were excluded from the analysis of other variables related to sexual behavior.

The “survey” module in the Stata 14 software was used for statistical analysis considering the complex sampling design (stratum, cluster and individual weight) to obtain population estimates.

### Ethical Considerations

Participation in the study was voluntary, and students had the option of not responding. No information that could identify the student and the school was collected. PeNSE 2015 was approved by the National Research Ethics Committee (Conep) through Opinion nº 1.006.467/201517.

## RESULTS

Females accounted for 51.3% (95%CI 50.7–51.9) and males for 48.7% (95%CI 48.1–49.3). With regard to age, 18.2% (95%CI 18.2–19.3) were between 11 and 13 years old, 78% (95%CI 76.9–79.0) were between 14 and 16 years old, and 3.8 % (95%CI 3.5–4.1) were aged 17 to 19 years.

In 2015, 27.5% of Brazilian adolescents enrolled in the 9th grade of elementary schools declared having had sexual initiation. Of these, 61.2% reported using a condom in the first intercourse and 68.6% in the last sexual intercourse, 44.0% reported using contraceptive methods, and the average number of partners reported was 2.8. Among males, higher rates of sexual initiation, non-use of condoms or contraceptive methods and a greater number of sexual partners were observed (Table 1).

**Table 1:** Prevalence of sexual behaviors among Brazilian adolescents according to sex. National Adolescent School-based Health Survey, Brazil, 2015.

Sexual behaviors		Total	Females	Males
		% (95%CI)	% (95%CI)	% (95%CI)
Sexual initiation (n= 101,566)				p<0.0001*
	Yes	27.5 (26.7–28.3)	19.6 (18.7–20.4)	35.9 (34.9–37.1)
	No	72.5 (71.7–73.3)	80.4 (79.6–81.3)	64.0 (63.0–65.1)
Used condom in 1st sexual intercourse (n=28,466)				p<0.0001*
	Yes	61.2(60.0–62.4)	68.7 (67.0–70.4)	56.8 (55.3–58.3)
	No	38.8 (37.6–40.0)	31.3 (29.6–33.0)	43.2 (41.7–44.7)
Used condom in the last sexual intercourse (n= 27,406)				p=0.019*
	Yes	68.6 (67.4–69.6)	66.9 (65.1–68.6)	69.6 (68.1–70.9)
	No	31.4 (30.4–32.6)	33.1 (31.4–34.9)	30.5 (29.1–31.9)
Used a contraceptive method in the last sexual intercourse (n= 24,863)				p<0.0001*
	Yes	44.0 (42.8–45.1)	46.8 (45.0–48.7)	42.1 (40.7–43.5)
	No	56.0 (54.9–57.2)	53.2 (51.3–55.0)	57.9 (56.5–59.3)
Number of partners (n= 28,401)				p<0.0001**
	Mean (±SE)	2.8 (2.7–2.8)	2.1 (2.0–2.1)	3.2 (3.2–3.3)

SE: standard error.

* Pearson's χ^2^ test

** Student's t test

As for parental supervision, the proportion of young people who reported missing classes without permission was higher among males (8.4%). Among males, there was also a higher proportion of parents who were not aware of their children's free-time activities (37.3%). A higher proportion of parents did not check homework (70.1%), did not understand concerns (58.9%) and were not frequently present at meals (29.2%) among female adolescents in comparison with male adolescents (66.1, 53.4 and 22.5%, respectively). When evaluating the parental supervision score, there was a higher prevalence of maximum supervision (score 5) among males (15.8%) (Table 2).

**Table 2:** Prevalence of parental supervision indicators and supervision score of Brazilian adolescents, according to sex. National Adolescent School-based Health Survey, Brazil, 2015.

Parental supervision indicators		Total	Females	Males
		% (95%CI)	% (95%CI)	% (95%CI)
Missing class without parental permission				p<0.001*
	3x or more in the last 30 days	7.7 (7.2–8.2)	7.1 (6.5–7.8)	8.4 (7.8–9.0)
	2x or less in the last 30 days	92.3 (91.8–92.7)	92.9 (92.2–93.5)	91.6 (31.0–92.2)
Parents aware of free-time activities				p<0.001*
	Rarely/never/sometimes	33.9 (33.2–34.5)	30.6 (29.8–31.4)	37.3 (36.5–38.1)
	Mostly/Always	66.1 (65.5–66.8)	69.4 (68.6–70.2)	62.7 (61.9–63.5)
Parents check that homework has been done				p<0.001*
	Rarely/never/sometimes	68.2 (67.5–68.7)	70.1 (69.4–70.9)	66.1 (65.2–66.9)
	Mostly/Always	31.8 (31.2–32.4)	29.9 (29.1–30.6)	34.0 (33.1–34.8)
Parents understand their problems and concerns				p<0.001*
	Rarely/never/sometimes	56.2 (55.5–56.8)	58.9 (58.1–59.7)	53.4 (52.4–54.4)
	Mostly/Always	43.8 (43.1–44.4)	41.1 (40.3–41.9)	46.6 (45.7–47.6)
Parents present at meals often				p<0.001*
	4x or less during the week	26.0 (25.3–26.6)	29.2 (28.4–30.0)	22.5 (21.7–23.3)
	5x or more during the week	74.0 (73.4–74.7)	70.8 (70.0–71.6)	77.5 (76.7–78.3)
Supervision scorer				p<0.001*
	0	1.3 (1.1–1.5)	1.4 (1.2–1.7)	1.1 (1.0–1.3)
	1	9.1 (8.8–9.5)	9.7 (9.3–10.2)	8.5 (8.1–9.0)
	2	22.7 (22.2–23.2)	22.6 (22.0–23.3)	22.7 (22.0–23.5)
	3	28.6 (28.1–29.1)	29.5 (28.8–30.2)	27.7 (26.9–28.4)
	4	23.6 (23.1–24.1)	23.1 (22.5–23.7)	24.2 (23.5–25.0)
	5	14.7 (14.2–15.1)	13.7 (13.1–14.2)	15.8 (15.1–16.4)

* Pearson's χ^2^ test.

The greater the parental supervision, the lower the prevalence of sexual initiation and the greater the prevalence of protective sexual behaviors. Of the adolescents with the lowest supervision score, 58% had had sexual initiation, against 20.1% of those with greater parental supervision. Adolescents under high supervision (score 5) used condoms more frequently in the very first (71.1%) and last sexual intercourse (80.2%), while adolescents who had minimal supervision had a prevalence of 49.8 and 50.9%, respectively. With regard to the use of contraceptive methods, the prevalence is also higher when under strict supervision (49.1 against 40.8% of adolescents under low supervision). The number of sexual partners was higher among those under low supervision (mean of 3.25 versus 2.88 for those with high supervision) (Table 3 and Supplementary Table 1).

**Table 3:** Prevalence of sexual behaviors among male and female Brazilian adolescent students according to parental supervision indicators. National Adolescent School-based Health Survey, Brazil, 2015.

Parental supervision indicators		Sexual initiation	Condom in first intercourse	Condom in last intercourse	CM in last intercourse	N. of partners
		% (95%CI)	% (95%CI)	% (95%CI)	% (95%CI)	Mean (95%CI)
Missing class without parental permission						
	3x or more in the last 30 days	49.3 (47.1–51.6)*	54.1 (51.1–57.1)*	58.9 (55.8–62.0)*	43.1 (39.3–47.1)	3.18 (3.04–3.33)
	2x or less in the last 30 days	25.7 (24.9–26.4)	62.3 (61.0–63.5)	70.0 (68.9–71.2)	44.1 (42.9–45.4)	2.73 (2.68–2.77)
Parents aware of free-time activities						
	Rarely/never/sometimes	38.4 (37.2–39.6)*	57.8 (56.2–59.5)*	65.6 (64.0–67.2)*	44.4 (42.7–46.0)	2.92 (2.86–2.98)
	Mostly/Always	21.9 (21.1–22.7)	64.1 (62.6–65.5)	71.1 (69.6–72.5)	43.7 (42.2–45.2)	2.67 (2.68–2.73)
Parents check that homework has been done						
	Rarely/never/sometimes	28.3 (27.4–29.2)*	58.3 (56.8–59.6)*	64.9 (63.6–66.2)*	41.8 (40.4–43.2)*	2.76 (2.71–2.81)
	Mostly/Always	25.7 (24.8–26.7)	67.8 (65.9–69.6)	76.8 (75.0–78.5)	49.3 (47.3–51.2)	2.86 (2.79–2.93)
Parents understand their problems and concerns						
	Rarely/never/sometimes	30.0 (29.0–30.9)*	58.4 (56.9–59.9)*	65.3 (63.8–66.6)*	41.6(40.1–43.1)*	2.75 (2.69–2.80)
	Mostly/Always	24.3 (23.3–25.2)	65.5 (63.9–67.1)	73.8 (72.2–75.3)	47.8 (46.1–49.5)	2.85 (2.80–2.91)
Parents present at meals often						
	4x or less during the week	32.1 (30.9–33.2)*	58.2 (56.4–59.9)*	64.1 (62.2–65.8)*	43.3 (41.4–45.2)	2.77 (2.69–2.85)
	5x or more during the week	25.9 (25.0–26.8)	62.5 (61.0–63.9)	70.5 (69.2–71.7)	44.3 (43.0–45.7)	2.80 (2.75–2.85)

* P value of Pearson's χ^2^ test <0.001; % population estimates; 95%CI: 95% confidence interval; CM: contraceptive method; N.: absolute number;

Table 4 and Supplementary Table 2 show the crude and age-adjusted PRs between adolescents’ sexual behaviors and the parental supervision score. It was observed that the higher the parental supervision score, the lower the prevalence of sexual initiation (p<0.001), the higher the prevalence of contraceptive methods and condom use in the first (p<0.001) and last sexual intercourse (p< 0.001), and the lower the average number of partners (p<0.05), even after adjustments.

**Table 4:** Prevalence ratios and unadjusted and adjusted β-coefficient and 95% confidence intervals of the sexual behaviors of adolescent Brazilian schoolchildren according to parental supervision. National Adolescent School-based Health Survey, Brazil, 2015.

Parental supervision		Sexual initiation	Condom in first intercourse	Condom in last intercourse	CM in last intercourse	N. of partners (β)
Supervision score (crude analysis)						
	0	Ref.	Ref.	Ref.	Ref.	Ref.
	1	0.70 (0.63–0.78)	1.07 (0.92–1.26)	1.22 (1.04–1.43)	1.00 (0.83–1.23)	-0.37 (-0.69;-0.06)
	2	0.57 (0.52–0.63)	1.16 (1.00–1.34)	1.24 (1.06–1.45)	1.03 (0.86–1.24)	-0.41 (-0.72;-0.09)
	3	0.44 (0.39–0.49)	1.24 (1.07–1.44)	1.35 (1.16–1.58)	1.07 (0.88–1.29)	-0.51 (-0.81;-0.21)
	4	0.37 (0.33–0.41)	1.34 (1.15–1.56)	1.49 (1.28–1.74)	1.16 (0.96–1.40)	-0.54 (-0.84;-0.25)
	5	0.34 (0.31–0.39)	1.43 (1.22–1.66)	1.57 (1.35–1.84)	1.20 (0.99–1.46)	-0.35 (-0.66;-0.04)
Supervision score (adjusted analysis)*						
	0	Ref.	Ref.	Ref.	Ref.	Ref.
	1	0.75 (0.68–0.84)	1.08 (0.93–1.27)	1.22 (1.04–1.43)	1.03 (0.84–1.25)	-0.40 (-0.69;-0.10)
	2	0.62 (0.56–0.69)	1.19 (1.03–1.38)	1.24 (1.06–1.45)	1.06 (0.88–1.28)	-0.50 (-0.80;-0.20)
	3	0.52 (0.47–0.58)	1.28 (1.10–1.48)	1.35 (1.16–1.59)	1.10 (0.90–1.33)	-0.59 (-0.88;-0.30)
	4	0.44 (0.39–0.48)	1.39 (1.20–1.61)	1.49 (1.28–1.74)	1.20 (0.99–1.45)	-0.66 (-0.94;-0.38)
	5	0.40 (0.36–0.45)	1.50 (1.29–1.75)	1.57 (1.34–1.84)	1.25 (1.03–1.53)	-0.54 (-0.84;-0.24)

* P value of Pearson's χ^2^ test <0.001; CM: contraceptive method; N.: absolute number;

## DISCUSSION

The findings of this study showed gender inequality in the way parents supervise their children, since male adolescents are less supervised in activities outside their homes, and female adolescents are less supervised in the home environment. However, a review of the moderating role of gender in the relationship between parenting and adolescent sexual risk behavior suggests that parental monitoring may be more protective for male adolescents with regard to sexual risk behavior, while for female adolescents, the emotional connection with their parents may be a more important factor in preventing risky sexual behavior^10^. The difference found in this study in the way parents supervise adolescents according to gender may be influenced by social models for men and women that are still present in society, since, throughout history, women occupied the domestic space and men the public and political spaces^19^. Despite this uneven pattern, the protective effect of parental supervision was similar for both girls and boys (data not shown).

Sexual initiation was more prevalent among male adolescents, and previous studies show that male adolescents indeed initiate sexual life earlier^4,9,20,21^. Gender aspects can also explain this difference in the age of sexual initiation, since male and female roles in traditional social representation guide different expectations, in which men's behaviors are related to masculinity and virility, while, for women, expectations are that they are more dependent, conformist and submissive^22^. Thus, parental monitoring differs according to biological sex^9^.

Our findings show that, among adolescents who have already started their sexual lives, health risk sexual behaviors were less frequent as the score of parental supervision increased. Adolescents under greater supervision showed greater use of condoms in the first and last sexual intercourse, greater use of contraceptive methods and fewer sexual partners. These results suggest that the presence of parents in the lives of adolescents can provide conditions for sharing information, advice and positive skills with adolescents^9^, representing an opportunity to address issues related to sexuality. It is added that this finding corroborates international studies^2,6–8^ and points out the role of parental educational practices that offer monitoring and application of rules at the same time they practice understanding and proximity attitudes as protection against the involvement of adolescents in behaviors that may pose a risk to sexual health, making them less susceptible to sexually transmitted infections (STIs) and pregnancy—outcomes that are increasing in our country among adolescents^23–26^.

Regarding STIs, a significant increase in the number of cases is reported^23,26^. In addition to these rates having increased in the last decade, another factor of great concern is the reduction in knowledge about the serological status in relation to the human immunodeficiency virus (HIV) and other STIs, especially among people aged 14 to 25^23^. These data, in view of our findings, make us reflect on the constant need for health actions aimed at preventing STIs.

Another important outcome in this scenario is teenage pregnancy, which can lead to consequences mainly in the lives of women, such as interruption of studies^16^. This, in turn, has a long-term impact on opportunities to complete education and enter the labor market, contributing to situations of greater vulnerability for these women and their children and for the perpetuation of the cycle of poverty and social exclusion^27^. In addition, teenage pregnancy reinforces gender inequalities. Including male adolescents in actions to reduce pregnancy is challenging, especially when considering the need to promote reflections on masculinity, virility, inequality and gender violence^27^. In this sense, the results of the study show that including the family in health actions, in order to strengthen the bond between parents and children, can be a promising strategy to reduce sexual risky behaviors among adolescents.

An American longitudinal study also reported that the proximity, monitoring and communication of parents during adolescence brought positive repercussions on the sexual health of their children, in addition to an impact on subsequent sexual behavior, after the teenage years^28^. It should also be noted that parental supervision was proven protective against other risky behaviors among adolescents, such as consumption of alcohol^29^, use of tobacco and drugs^30^, sedentary lifestyle and physical inactivity^31^. These findings reinforce the importance of the bond between parents and children, in the sense that familial practices such as supervision and parental presence can protect adolescents from negative health outcomes.

Other factors are known to also contribute to the sexual and reproductive behavior of adolescents, namely: schooling, use of licit and illicit drugs, peer influence, religion, dating relationships, access to health and education services, among others^8,32^. It should be added that, in addition to the family, the school and health services must play a strategic role in the training and education of children and adolescents, and must be understood as a central factor in the network for promoting the health of children and young people^2,3^. For this, intersectoral initiatives between education and health, such as the Health at School Program (PSE in the Brazilian acronym), are essential for a comprehensive health care for students. In this context, the professional nurse assumes the role by outlining effective actions for the adolescent public in order to build knowledge and start discussions, being able to contribute to better health practices together with parents and schools, to strengthen the bond and reduce risk behaviors among these young people^33^.

These programs and policies are essential to advance in the field of adolescent sexual and reproductive health, which was until recently a priority of primary care in the country^34^. Thus, this research is believed to contribute to the construction of the theme, in view of the uncertain scenario of public policies in this matter in the country, since some setbacks have been observed. Among them, the following stand out: our country's failure to sign the commitment of our country with the World Health Organization (WHO) regarding sexual and reproductive health of populations^35^; the withdrawal of the adolescents’ booklets from circulation, especially addressing information related to sex education and safe sex^36^; the encouragement of sexual abstinence policies, which have already been considered ineffective^37^; budget cuts^18^; and several other setbacks previously described, which can support the argument of a possible programmatic emptying of sexual and reproductive health in the country^17^.

This study has some limitations, such as the inclusion of only adolescents who attended school, since data from the IBGE^38^ indicate that 15.0% of adolescents aged 15 to 17 years were out of school in 2015. Another limitation is that it uses a parental monitoring proxy measure, not being able to address all aspects related to parental supervision. The different chances of sexual initiation at different ages are also a limit to be considered, which was corrected by adjusting the estimates for this variable.

It is important to highlight that the score added supervision of different natures, such limits/responsibilities and dialogue and affection. However, we found a relation that suggests dose-response: the greater the supervision, the lesser the behaviors considered risky, which may mean that both elements are important in parental supervision.

Even with the release of the PeNSE 2019 macro data, the difficulty in accessing the databases of this research on the health of adolescents in Brazil prevents the most up-to-date assessment of these indicators and this relationship over time. Finally, the pandemic is believed to have caused major changes in parental supervision and adolescent sexual and reproductive health, which requires further investigation.

Despite these limitations, as it is a nationally representative survey that interviewed more than 100 thousand adolescents, the findings of this study are of great relevance for the country, since few studies have comprehensively investigated parental supervision and adolescent sexual behavior.

This investigation showed that adolescents who are under greater supervision by their parents have more responsible sexual behaviors, even though gender inequalities regarding parental supervision have been observed. The results show the fundamental role of the family in providing adolescents with conditions that stimulate healthy and risk-free sexual behavior through monitoring along with dialogue and affection.
