# Supplementary material for: Parental supervision and sexual behavior among Brazilian adolescents
Source: Rev Bras Epidemiol. 2023 Apr 21;26(Suppl 1):e230013. doi: 10.1590/1980-549720230013.supl.1 (PMC10176735; doi:10.1590/1980-549720230013.supl.1)
Supplement: Supplementary file 1 [file 1980-5497-rbepid-26-suppl1-e230013-suppl1.pdf]

Tabela Suplementar 1. Prevalência dos comportamentos sexuais dos adolescentes escolares brasileiros de ambos os sexos conforme indicadores e escore supervisão. Pesquisa Nacional de Saúde do Escolar, Brasil, 2015.

**Indicadores de supervisão dos pais**	**Iniciação sexual**	**Uso de preservativo na primeira relação**	**Uso de preservativo na última relação**	**Uso de método contraceptivo na última relação**	**Número de parceiros sexuais**
	**% (IC 95%)**	**% (IC 95%)**	**% (IC 95%)**	**% (IC 95%)**	**Média (IC 95%)**
**Falta às aulas sem permissão dos pais**	p<0,001	p<0,001	p<0,001	p=0,645	
>3x nos últimos 30 dias	49,3 (47,1-51,6)	54,1 (51,1-57,1)	58,9 (55,8-62,0)	43,1 (39,3-47,1)	3,18 (3,04-3,33)
Raramente/nunca	25,7 (24,9-26,4)	62,3 (61,0-63,5)	70,0 (68,9-71,2)	44,1 (42,9-45,4)	2,73 (2,68-2,77)
**Pais cientes das atividades no tempo livre**	p<0,001	p<0,001	p<0,001	p=0,527	
Raramente/ nunca	38,4 (37,2-39,6)	57,8 (56,2-59,5)	65,6 (64,0-67,2)	44,4 (42,7-46,0)	2,92 (2,86-2,98)
Na maior parte/sempre	21,9 (21,1-22,7)	64,1 (62,6-65,5)	71,1 (69,6-72,5)	43,7 (42,2-45,2)	2,67 (2,68-2,73)
**Pais verificam os deveres de casa**	p<0,001	p<0,001	p<0,001	p<0,001	
Raramente/ nunca	28,3 (27,4-29,2)	58,3 (56,8-59,6)	64,9 (63,6-66,2)	41,8 (40,4-43,2)	2,76 (2,71-2,81)
Na maior parte / sempre	25,7 (24,8-26,7)	67,8 (65,9-69,6)	76,8 (75,0-78,5)	49,3 (47,3-51,2)	2,86 (2,79-2,93)
**Pais entenderam seus problemas e preocupações**	p<0,001	p<0,001	p<0,001	p<0,001	
Raramente/ nunca	30,0 (29,0-30,9)	58,4 (56,9-59,9)	65,3 (63,8-66,6)	41,6(40,1-43,1)	2,75 (2,69-2,80)
Na maior parte / sempre	24,3 (23,3-25,2)	65,5 (63,9-67,1)	73,8 (72,2-75,3)	47,8 (46,1-49,5)	2,85 (2,80-2,91)
**Pais presentes nas refeições frequentemente**	p<0,001	p<0,001	p<0,001	p=0,373	
Raramente/ nunca	32,1 (30,9-33,2)	58,2 (56,4-59,9)	64,1 (62,2-65,8)	43,3 (41,4-45,2)	2,77 (2,69-2,85)
Na maior parte / sempre	25,9 (25,0-26,8)	62,5 (61,0-63,9)	70,5 (69,2-71,7)	44,3 (43,0-45,7)	2,80 (2,75-2,85)
**Escore Supervisão**	p<0,001	p<0,001	p<0,001	p<0,001	
0	58,0 (52,4-63,5)	49,8 (42,6-57,0)	50,9 (43,2-58,5)	40,8 (33,4-48,5)	3,25 (2,93-3,25)
1	40,9 (39,0-42,8)	53,5 (50,7-56,3)	62,1 (59,1-65,0)	41,2 (38,3-44,1)	2,86(2,74-2,97)
2	33,2 (31,8-34,6)	57,7 (55,7-59,7)	63,3 (61,2-65,3)	42,1 (40,0-44,3)	2,82 (2,73-2,90)
3	25,8 (24,7-27,0)	61,7 (59,6-63,8)	69,0 (66,9-71,1)	43,6 (41,4-45,7)	2,72 (2,64-2,80)
4	21,6 (20,6-22,6)	66,8 (64,6-68,9)	76,0 (74,0-77,9)	47,4 (44,9-49,8)	2,68 (2,60-2,76)
5	20,1 (18,9-28,2)	71,1 (68,1-73,9)	80,2 (77,6-82,6)	49,1 (45,9-52,3)	2,88 (2,76-3,00)

Nota: Valor-p do teste qui-quadrado de Pearson; % estimativas populacionais; IC95%=Intervalo de 95% de confiança.

Tabela Suplementar 2 - Razões de prevalência (RP) não ajustadas e ajustadas e intervalos de confiança de 95% (IC95%) dos comportamentos sexuais dos adolescentes escolares brasileiros de ambos os sexos conforme supervisão dos pais. Pesquisa Nacional de Saúde do Escolar, Brasil, 2015.

Indicadores de supervisão dos pais	Iniciação sexual	Uso de preservativo na primeira relação	Uso de preservativo na última relação	Uso de método contraceptivo na última relação	Número de parceiros sexuais
	RP (IC 95%)	RP (IC 95%)	RP (IC 95%)	RP (IC 95%)	Mean diff. (IC95%)
**Falta de aula sem permissão dos pais**					
Sim	Ref.	Ref.	Ref.	Ref.	Ref.
Não (não ajustada)	0,52 (0,49-0,54)	1,15 (1,08-1,22)	1,19 (1,12-1,25)	1,02 (0,93-1,13)	-0,45 (-0,61;-0,31)
Não (ajustada)	0,60 (0,57-0,63)	1,16 (1,09-1,23)	1,18 (1,12-1,25)	1,03 (0,94-1,14)	-0,48 (-0,62; -0,35)
**Pais cientes das atividades no tempo livre**					
Não	Ref.	Ref.	Ref.	Ref.	Ref.
Sim (não ajustada)	0,57 (0,55-0,59)	1,11(1,07-1,14)	1,08 (1,05-1,12)	0,98 (0,94-1,03)	-0,25 (-0,32; -0,17)
Sim (ajustada)	0,67 (0,64-0,69)	1,10 (1.07-1,14)	1,09 (1,05-1,12)	0,99 (0,94-1,03)	-0,16 (-0,23; -0,09)
**Pais verificam os deveres de casa**					
Não	Ref.	Ref.	Ref.	Ref.	Ref.
Sim (não ajustada)	0,91 (0,87-0,95)	1,16 (1,12-1,20)	1,18 (1,15-1,22)	1,18 (1,12-1,24)	0,10 (0,01; 0,18)
Sim (ajustada)*	0,90 (0,86-0,93)	1,18 (1,14-1,22)	1,18 (1,14-1,21)	1,19 (1,13-1,25)	0,003 (-0,08;0,08)
**Pais entenderam seus problemas e preocupações**					
Não	Ref.	Ref.	Ref.	Ref.	Ref.
Sim (não ajustada)	0,81 (0,78-0,84)	1,12 (1,09-1,16)	1,13 (1,10-1,16)	1,15 (1,09-1,21)	0,11 (0,03; 0,18)
Sim (ajustada)*	0,81 (0,78-0,84)	1,14 (1,10-1,80)	1,13 (1,09-1,16)	1,16 (1,10-1,22)	0,02 (-0,05; 0,09)
**Pais presentes nas refeições frequentemente**					
Não	Ref.	Ref.	Ref.	Ref.	Ref.
Sim (não ajustada)	0,81 (0,77-0,84)	1,07 (1,04-1,11)	1,10 (1,06-1,13)	1,02 (0,97-1,07)	0,03 (-0,07;0,12)
Sim (ajustada)*	0,80 (0,77-0,83)	1,10 (1,07-1,15)	1,09 (1,06-1,13)	1,04 (0,99-1,10)	- 0,10 (-0,20;-0,01)
**Escore Supervisão (não ajustada)**					
0	Ref.	Ref.	Ref.	Ref.	Ref.
1	0,70 (0,63-0,78)	1,07 (0,92-1,26)	1,22 (1,04-1,43)	1,00 (0,83-1,23)	-0,37 (-0,69;-0,06)
2	0,57 (0,52-0,63)	1,16 (1,00-1,34)	1,24 (1,06-1,45)	1,03 (0,86-1,24)	-0,41 (-0,72;-0,09)
3	0,44 (0,39-0,49)	1,24 (1,07-1,44)	1,35 (1,16-1,58)	1,07 (0,88-1,29)	-0,51 (-0,81;-0,21)
4	0,37 (0,33-0,41)	1,34 (1,15-1,56)	1,49 (1,28-1,74)	1,16 (0,96-1,40)	-0,54 (-0,84;-0,25)
5	0,34 (0,31-0,39)	1,43 (1,22-1,66)	1,57 (1,35-1,84)	1,20 (0,99-1,46)	-0,35 (-0,66;-0,04)
**Escore Supervisão (ajustada)***					
0	Ref.	Ref.	Ref.	Ref.	Ref.
1	0,75 (0,68-0,84)	1,08 (0,93-1,27)	1,22 (1,04-1,43)	1,00 (0,83-1,23)	-0,40 (-0,69;-0,10)
2	0,62 (0,56-0,69)	1,19 (1,03-1,38)	1,24 (1,06-1,45)	1,03 (0,86-1,24)	-0,50 (-0,80;-0,20)
3	0,52 (0,47-0,58)	1,28 (1,10-1,48)	1,35 (1,16-1,59)	1,07 (0,88-1,29)	-0,59 (-0,88;-0,30)
4	0,44 (0,39-0,48)	1,39 (1,20-1,61)	1,49 (1,28-1,74)	1,16 (0,96-1,40)	-0,66 (-0,94;-0,38)
5	0,40 (0,36-0,45)	1,50 (1,29-1,75)	1,57 (1,34-1,84)	1,20 (0,99-1,46)	-0,54 (-0,84;-0,24)

Nota: Ref.= Referência. *= ajustado por sexo e idade; RP= Razão de Prevalência; Mean diff.=diferença de médias; IC95% Intervalo de 95% de confiança.
