# Supplementary material for: Parental supervision and sexual behavior among Brazilian adolescents
Source: Rev Bras Epidemiol. 2023 Apr 21;26(Suppl 1):e230013. doi: 10.1590/1980-549720230013.supl.1 (PMC10176735; doi:10.1590/1980-549720230013.supl.1)
Supplement: Translation

# Translation: Supervisão dos pais e comportamento sexual entre adolescentes brasileiros

**DOI:** 10.1590/1980-549720230013.supl.1.1

**Authors:** Gleice Barbosa Reis, Marco Aurélio de Sousa, Gisele Nepomuceno de Andrade, Deborah Carvalho Malta, Ísis Eloah Machado, Mariana Santos Felisbino-Mendes

**Affiliations:** I Universidade Federal de Minas Gerais, Escola de Enfermagem – Belo Horizonte (MG), Brasil.; II Universidade Federal de Minas Gerais, Escola de Enfermagem, Departamento de Enfermagem Materno-Infantil e Saúde Pública, Programa de Pós-Graduação em Enfermagem – Belo Horizonte (MG), Brasil.; III Universidade Federal de Ouro Preto, Departamento de Medicina de Família, Saúde Mental e Coletiva, Programa de Pós-Graduação em Saúde e Nutrição – Ouro Preto (MG), Brasil.

**Keywords:** Sexo sem proteção, Planejamento familiar, Adolescente, Relações pais-filho, Saúde sexual e reprodutiva, Iniquidade de gênero

## Abstract

**Objetivo:**

Avaliar a associação entre a supervisão dos pais e comportamentos sexuais entre os adolescentes brasileiros.

**Métodos:**

Estudo transversal com dados de 102.072 estudantes do 9º ano que responderam à Pesquisa Nacional de Saúde do Escolar de 2015. Estimou-se a prevalência dos comportamentos sexuais (iniciação, uso de preservativo, contracepção e número de parcerias). A supervisão dos pais foi avaliada por meio de escore formado por cinco indicadores. Foram calculadas razões de prevalência ajustadas por sexo e idade para a análise das relações existentes entre o escore de supervisão dos pais e os comportamentos sexuais de adolescentes.

**Resultados:**

As prevalências de comportamentos sexuais em adolescentes com mínima e máxima supervisão parental foram: iniciação sexual (mín.: 58,0%; máx.: 20,1%), uso do preservativo na última relação sexual (mín.: 50,9%; máx.: 80,2%), de contraceptivos (mín.: 40,8; máx.: 49,1%) e número de parceiros (mín.: 3,25; máx.: 2,88). A supervisão parental apresentou maior magnitude no sexo feminino. Aqueles com maior escore de supervisão apresentaram maiores prevalências do uso de preservativos na primeira e última relação sexual, de métodos contraceptivos e menor média do número de parceiros, mesmo após ajustes por sexo e idade.

**Conclusão:**

Quanto maior a supervisão dos pais, melhores os comportamentos sexuais, para ambos os sexos, apesar de a supervisão ocorrer de forma diferenciada entre os sexos. Esses achados apontam o papel da família em proporcionar aos adolescentes monitoramento simultâneo ao diálogo e ao afeto, condição estimuladora do comportamento sexual saudável e livre de riscos.

## INTRODUÇÃO

A adolescência é marcada por transformações corporais, cognitivas, emocionais e sociais, que por sua vez motivam transformações dos padrões de comportamento e demandam maior atenção familiar^1,2^. Sabe-se que as possibilidades de aprendizagem, descobertas e experimentações dessa faixa etária podem conduzir a situações de risco e vulnerabilidade, especialmente quando relacionadas à sexualidade^3^. As práticas sexuais inseguras ocorrem principalmente em razão da existência de tabus na descoberta e exercício da sexualidade, da falta de informações e da ausência de comunicação com familiares^4^.

Com relação à família, a supervisão parental, um tipo de monitoramento indireto do comportamento dos adolescentes^5^ que inclui a aplicação de regras e o conhecimento dos pais sobre a localização e as atividades dos filhos, bem como o diálogo e o afeto, entre outros fatores, pode contribuir para modificar o comportamento sexual entre os adolescentes^2,6^. Estudos prévios demonstraram que níveis mais altos desse monitoramento dos pais estão relacionados à maior probabilidade de retardar a iniciação sexual^6^ e ao uso de preservativos e contraceptivos^2,7,8^, contribuindo para a adoção de práticas saudáveis com relação à saúde sexual e reprodutiva pelos adolescentes. Ademais, observou-se o efeito protetor da supervisão dos pais em outros desfechos de saúde^2,6,7,9^.

Estudos internacionais^2,6,10^ de âmbito nacional^11,12,13^, regional^1,4,14,15^ e local, como em população rural^16^, têm cada vez mais investigado o comportamento sexual dos adolescentes, incluindo a relação com a supervisão parental. Entretanto, pesquisas que consideram as diferenças de gênero são menos frequentes e abordam poucos indicadores de supervisão parental^8^, geralmente relacionados ao conhecimento sobre a localização do adolescente quando não está na escola, o que remete à supervisão relativa a limites e responsabilidades. No entanto, questões que remetem ao diálogo e ao afeto também devem ser consideradas^10^. Um estudo de revisão mostrou diferenças na supervisão a depender do gênero, com meninos recebendo maior supervisão dos comportamentos sexuais de risco enquanto meninas recebem mais suporte emocional^10^.

Nesse sentido, o presente estudo pretendeu avaliar a associação entre a supervisão dos pais e os comportamentos sexuais adotados pelos adolescentes brasileiros de ambos os sexos. Tem-se como hipótese que adolescentes com maior supervisão dos pais apresentam comportamentos sexuais de menor risco.

## MÉTODO

Trata-se de estudo transversal que analisou dados da Amostra 1 da Pesquisa Nacional de Saúde do Escolar (PeNSE) realizada em 2015, composta de 102.072 adolescentes escolares matriculados e que frequentavam regularmente escolas públicas e privadas situadas nas zonas urbanas e rurais de todo o território nacional no ano letivo de 2015^17^. A pesquisa foi realizada pelo Ministério da Saúde (MS) em parceria como Instituto Brasileiro de Geografia e Estatística (IBGE) e o Ministério da Educação (MEC).

### Desenho amostral e coleta de dados

A Amostra 1 da PeNSE 2015 foi planejada para estimar parâmetros populacionais do Brasil, Grandes Regiões, Unidades da Federação e municípios das capitais^17^. Para o plano amostral foram definidos 53 estratos geográficos. Cada uma das 26 capitais estaduais, mais o Distrito Federal, foi definida como um estrato geográfico, e os demais municípios foram agrupados em mais 26 estratos representando cada um dos estados brasileiros, excluídas as capitais^17^. Em cada um dos 53 estratos formados, foi dimensionada e selecionada uma amostra de escolas com base no cadastro constituído por informações do Censo Escolar de 2013^17^. Para garantir a presença de escolas públicas (federais, estaduais ou municipais) e privadas em proporção aproximada à de sua existência no cadastro de seleção, foram formados estratos de alocação pelo cruzamento dos estratos geográficos, com a dependência administrativa das escolas (pública ou privada) e o tamanho delas, medido pelo número de turmas de 9º ano das escolas^17^.

Em cada escola da amostra, as turmas do 9º ano foram selecionadas por sorteio, e os respectivos alunos presentes no dia da coleta de dados foram convidados a responder, por meio de um *smartphone*, ao questionário da pesquisa. Este era dividido em módulos temáticos sobre diversos fatores de risco e proteção, incluindo questões sobre a supervisão dos pais recebida e sobre a saúde sexual e reprodutiva^17^. Outras informações sobre o plano amostral da PeNSE e a metodologia de coleta de dados podem ser consultadas em: https://www.ibge.gov.br/estatisticas/sociais/educacao/9134-pesquisa-nacional-de-saude-do-escolar.html?=&t=o-que-e

### Variáveis

A supervisão parental foi a variável de interesse deste estudo. As cinco perguntas utilizadas para avaliar de forma *proxy* a supervisão parental encontram-se no Quadro 1. Além da avaliação de cada variável, somaram-se as respostas das cinco variáveis gerando um escore, que variou de 0 a 5. Assim, os adolescentes que obtiveram escore igual a 0 (“sim” para a variável que analisa a falta às aulas sem a permissão dos pais e “não” para as outras variáveis) foram considerados com pouca ou nenhuma supervisão parental, e aqueles com escore 5 (“não” para a variável que analisa a falta às aulas dos adolescentes sem a permissão dos pais e “sim” para as outras variáveis) foram classificados como em intensa supervisão.

**Quadro 1:** Variáveis de supervisão parental e categorização.

Variáveis	Pergunta	Categorização	Pontuação
Falta de aula sem permissão dos pais	NOS ÚLTIMOS 30 DIAS, em quantos dias você faltou às aulas ou à escola sem permissão dos seus pais ou responsáveis?	Sim — 3 dias ou mais nos últimos 30 dias	0
		Não — 2 dias ou menos nos últimos 30 dias	1
Pais cientes das atividades no tempo livre	NOS ÚLTIMOS 30 DIAS, com que frequência seus pais ou responsáveis sabiam realmente o que você estava fazendo em seu tempo livre?	Sim — na maior parte das vezes ou sempre	1
		Não — nunca, raramente ou às vezes	0
Pais verificam se os deveres de casa foram feitos	NOS ÚLTIMOS 30 DIAS, com que frequência seus pais ou responsáveis verificaram se os seus deveres de casa (lição de casa) foram feitos?	Sim — a maior parte do tempo ou sempre	1
		Não — nunca, raramente ou às vezes	0
Pais entenderam seus problemas e preocupações	NOS ÚLTIMOS 30 DIAS, com que frequência seus pais ou responsáveis entenderam seus problemas e preocupações?	Sim — na maior parte do tempo ou sempre	1
		Não — nunca, raramente ou às vezes	0
Pais presentes nas refeições frequentemente	Você costuma almoçar ou jantar com sua mãe, pai ou responsável?	Sim — 5 ou mais dias durante a semana	1
		Não — 4 ou menos dias durante a semana	0

O comportamento sexual entre os adolescentes foi avaliado por meio das seguintes variáveis: relação sexual alguma vez na vida (iniciação); usar preservativo na primeira relação sexual, usar preservativo na última relação sexual, usar método para evitar gravidez na última relação e número de parceiros na vida.

As demais covariáveis estudadas foram sexo (masculino, feminino) e faixa etária (11–13, 14–16, 17–19).

### Análise de dados

Estimou-se a prevalência dos indicadores de comportamento sexual entre os adolescentes brasileiros de acordo com a supervisão parental recebida. Em seguida, estimou-se a prevalência das variáveis de supervisão parental dos adolescentes segundo o sexo. Calculou-se ainda a média de parceiros sexuais informados pelos adolescentes, conforme a supervisão parental, em ambos os sexos. As diferenças estatísticas foram mensuradas pelo teste do χ^2^ de Pearson, com nível de significância de 5%, e pelos intervalos de confiança de 95% (IC95%).

Finalmente, foram estimadas as razões de prevalência (RP) não ajustadas e ajustadas por sexo e idade dos comportamentos sexuais dos adolescentes de acordo com o escore de supervisão parental, utilizando a regressão de Poisson, tendo em vista a magnitude da prevalência dos comportamentos sexuais^18^. Para a variável número de parceiros sexuais, foram realizadas regressões lineares não ajustada e ajustada para estimar as diferenças de médias. Para todas as análises foram estimados os IC95%.

Os adolescentes que afirmaram não ter iniciado relações sexuais (n=72.989) foram excluídos da análise das demais variáveis referentes aos comportamentos sexuais.

Utilizou-se o módulo *survey* para análises estatísticas considerando-se o desenho amostral complexo (estrato, conglomerado e peso do indivíduo) para a obtenção de estimativas populacionais por meio do *software* Stata 14.

### Considerações éticas

A participação no estudo foi voluntária, e o estudante tinha a possibilidade de não responder. Não foram coletadas informações que pudessem identificar o aluno e a escola. A PeNSE 2015 foi aprovada na Comissão Nacional de Ética em Pesquisa (Conep) por meio do Parecer nº 1.006.467/2015^17^.

## RESULTADOS

O sexo feminino correspondeu a 51,3% (IC95% 50,7–51,9) e o masculino a 48,7% (IC95% 48,1–49,3). Com relação à idade, 18,2% (IC95% 18,2–19,3) tinham entre 11 e 13 anos, 78% (IC95% 76,9–79,0) entre 14 e 16 anos, e 3,8% (IC95% 3,5–4,1) de 17 a 19 anos.

No ano de 2015, 27,5% dos adolescentes brasileiros matriculados no 9º ano do ensino fundamental declararam ter tido iniciação sexual. Deles, 61,2% usaram preservativo na primeira e 68,6% na última relação sexual, 44,0% fizeram uso de métodos contraceptivos, e a média do número de parceiros foi 2,8. No sexo masculino, observaram-se maiores taxas de iniciação sexual, não uso de preservativos ou contraceptivo e maior número de parceiros sexuais (Tabela 1).

**Tabela 1:** Prevalência dos comportamentos sexuais entre adolescentes escolares brasileiros de acordo com o sexo. Pesquisa Nacional de Saúde do Escolar, Brasil, 2015.

Comportamentos sexuais		Total	Feminino	Masculino
		% (IC95%)	% (IC95%)	% (IC95%)
Iniciação sexual (n= 101.566)				p<0,0001*
	Sim	27,5 (26,7–28,3)	19,6 (18,7–20,4)	35,9 (34,9–37,1)
	Não	72,5 (71,7–73,3)	80,4 (79,6–81,3)	64,0 (63,0–65,1)
Usou preservativo na 1ª relação sexual (n=28.466)				p<0,0001*
	Sim	61,2(60,0–62,4)	68,7 (67,0–70,4)	56,8 (55,3–58,3)
	Não	38,8 (37,6–40,0)	31,3 (29,6–33,0)	43,2 (41,7–44,7)
Usou preservativo na última relação sexual (n= 27.406)				p=0,019*
	Sim	68,6 (67,4–69,6)	66,9 (65,1–68,6)	69,6 (68,1–70,9)
	Não	31,4 (30,4–32,6)	33,1 (31,4–34,9)	30,5 (29,1–31,9)
Usou método contraceptivo na última relação sexual (n= 24.863)				p<0,0001*
	Sim	44,0 (42,8–45,1)	46,8 (45,0–48,7)	42,1 (40,7–43,5)
	Não	56,0 (54,9–57,2)	53,2 (51,3–55,0)	57,9 (56,5–59,3)
Nº de parceiros (n= 28.401)				p<0,0001**
	Média (±EP)	2,8 (2,7–2,8)	2,1 (2,0–2,1)	3,2 (3,2–3,3)

EP: erro padrão.

* Teste do χ^2^ de Pearson

** Teste *t* de Student

Quanto à supervisão dos pais, a proporção de jovens que relataram faltar às aulas sem permissão foi maior entre o sexo masculino (8,4%). No sexo masculino, também se observou maior proporção de pais que não eram cientes das atividades dos filhos no tempo livre (37,3%). Obteve-se maior proporção de pais que não verificavam os deveres de casa (70,1%), não entendiam as preocupações (58,9%) e não estavam frequentemente presentes nas refeições (29,2%) entre adolescentes do sexo feminino em comparação com adolescentes do sexo masculino (66,1, 53,4 e 22,5%, respectivamente). Ao se avaliar o escore de supervisão parental, averiguou-se maior prevalência de supervisão máxima (escore 5) no sexo masculino (15,8%) (Tabela 2).

**Tabela 2:** Prevalência de indicadores de supervisão e escore supervisão dos pais de adolescentes escolares brasileiros, de acordo com o sexo. Pesquisa Nacional de Saúde do Escolar, Brasil, 2015.

Indicadores de supervisão dos pais		Total	Feminino	Masculino
		% (IC95%)	% (IC95%)	% (IC95%)
Falta às aulas sem permissão dos pais				p<0,001*
	3x ou mais nos últimos 30 dias	7,7 (7,2–8,2)	7,1 (6,5–7,8)	8,4 (7,8–9,0)
	2x ou menos nos últimos	92,3 (91,8–92,7)	92,9 (92,2–93,5)	91,6 (31,0–92,2)
Pais cientes das atividades no tempo livre				p<0,001*
	Raramente /nunca/às vezes	33,9 (33,2–34,5)	30,6 (29,8–31,4)	37,3 (36,5–38,1)
	Na maior parte /sempre	66,1 (65,5–66,8)	69,4 (68,6–70,2)	62,7 (61,9–63,5)
Pais verificam os deveres de casa				p<0,001*
	Raramente /nunca /às vezes	68,2 (67,5–68,7)	70,1 (69,4–70,9)	66,1 (65,2–66,9)
	Na maior parte /sempre	31,8 (31,2–32,4)	29,9 (29,1–30,6)	34,0 (33,1–34,8)
Pais entenderam seus problemas e preocupações				p<0,001*
	Raramente /nunca /às vezes	56,2 (55,5–56,8)	58,9 (58,1–59,7)	53,4 (52,4–54,4)
	Na maior parte /sempre	43,8 (43,1–44,4)	41,1 (40,3–41,9)	46,6 (45,7–47,6)
Pais presentes nas refeições frequentemente				p<0,001*
	4x ou menos durante a semana	26,0 (25,3–26,6)	29,2 (28,4–30,0)	22,5 (21,7–23,3)
	5x ou mais durante a semana	74,0 (73,4–74,7)	70,8 (70,0–71,6)	77,5 (76,7–78,3)
Escore Supervisão				p<0,001*
	0	1,3 (1,1–1,5)	1,4 (1,2–1,7)	1,1 (1,0–1,3)
	1	9,1 (8,8–9,5)	9,7 (9,3–10,2)	8,5 (8,1–9,0)
	2	22,7 (22,2–23,2)	22,6 (22,0–23,3)	22,7 (22,0–23,5)
	3	28,6 (28,1–29,1)	29,5 (28,8–30,2)	27,7 (26,9–28,4)
	4	23,6 (23,1–24,1)	23,1 (22,5–23,7)	24,2 (23,5–25,0)
	5	14,7 (14,2–15,1)	13,7 (13,1–14,2)	15,8 (15,1–16,4)

* Teste do χ^2^ de Pearson.

Percebeu-se que, quanto maior a supervisão dos pais, menores foram as prevalências de iniciação sexual e maiores as de comportamentos sexuais protetores. Dos adolescentes com o menor escore de supervisão, 58% apresentaram iniciação sexual, contra 20,1% dos que possuíam maior supervisão dos pais. Os adolescentes com alta supervisão (escore 5) utilizaram com maior frequência preservativos na primeira (71,1%) e na última relação sexual (80,2%), enquanto os adolescentes que tiveram o mínimo de supervisão apresentaram prevalências de 49,8 e 50,9%, respectivamente. No que tange ao uso de métodos contraceptivos, também se vê maior prevalência na presença de alta supervisão (49,1 contra 40,8% dos adolescentes com baixa supervisão). O número de parceiros sexuais foi maior entre aqueles com baixa supervisão (média de 3,25 contra 2,88 de quem tem alta supervisão) (Tabela 3 e Tabela Suplementar 1).

**Tabela 3:** Prevalência dos comportamentos sexuais dos adolescentes escolares brasileiros de ambos os sexos conforme indicadores de supervisão. Pesquisa Nacional de Saúde do Escolar, Brasil, 2015.

Supervisão dos pais		Iniciação sexual	Preserv. primeira	Preserv. última	Usou MC última	Nº de parceiros
		% (IC95%)	% (IC95%)	% (IC95%)	% (IC95%)	Média (IC95%)
Falta às aulas sem permissão dos pais						
	3x ou mais nos últimos 30 dias	49,3 (47,1–51,6)*	54,1 (51,1–57,1)*	58,9 (55,8–62,0)*	43,1 (39,3–47,1)	3,18 (3,04–3,33)
	2x ou menos nos últimos 30 dias	25,7 (24,9–26,4)	62,3 (61,0–63,5)	70,0 (68,9–71,2)	44,1 (42,9–45,4)	2,73 (2,68–2,77)
Pais cientes das atividades no tempo livre						
	Raramente/nunca/às vezes	38,4 (37,2–39,6)*	57,8 (56,2–59,5)*	65,6 (64,0–67,2)*	44,4 (42,7–46,0)	2,92 (2,86–2,98)
	Na maior parte/sempre	21,9 (21,1–22,7)	64,1 (62,6–65,5)	71,1 (69,6–72,5)	43,7 (42,2–45,2)	2,67 (2,68–2,73)
Pais verificam os deveres de casa						
	Raramente/nunca/às vezes	28,3 (27,4–29,2)*	58,3 (56,8–59,6)*	64,9 (63,6–66,2)*	41,8 (40,4–43,2)*	2,76 (2,71–2,81)
	Na maior parte/sempre	25,7 (24,8–26,7)	67,8 (65,9–69,6)	76,8 (75,0–78,5)	49,3 (47,3–51,2)	2,86 (2,79–2,93)
Pais entenderam seus problemas e preocupações						
	Raramente/nunca/às vezes	30,0 (29,0–30,9)*	58,4 (56,9–59,9)*	65,3 (63,8–66,6)*	41,6(40,1–43,1)*	2,75 (2,69–2,80)
	Na maior parte/sempre	24,3 (23,3–25,2)	65,5 (63,9–67,1)	73,8 (72,2–75,3)	47,8 (46,1–49,5)	2,85 (2,80–2,91)
Pais presentes nas refeições frequentemente						
	4x ou menos durante a semana	32,1 (30,9–33,2)*	58,2 (56,4–59,9)*	64,1 (62,2–65,8)*	43,3 (41,4–45,2)	2,77 (2,69–2,85)
	5x ou mais durante a semana	25,9 (25,0–26,8)	62,5 (61,0–63,9)	70,5 (69,2–71,7)	44,3 (43,0–45,7)	2,80 (2,75–2,85)

* Valor p do teste do χ^2^ de Pearson <0,001; % estimativas populacionais; IC95%: intervalo de confiança de 95%; MC: método contraceptivo; Nº: número absoluto; Preserv.: uso de preservativo.

A Tabela 4 e a Tabela Suplementar 2 apresentam as RP não ajustada e ajustada por sexo e idade entre comportamentos sexuais dos adolescentes e o escore de supervisão parental. Observou-se que, quanto maior o escore de supervisão dos pais, menor a prevalência de iniciação sexual (p<0,001), maior a prevalência de uso de contraceptivos e preservativos na primeira (p<0,001) e na última relação sexual (p<0,001) e menor o número médio de parceiros (p<0,05), mesmo após ajustes.

**Tabela 4:** Razões de prevalência e coeficiente β não ajustados e ajustados e intervalos de confiança de 95% dos comportamentos sexuais dos adolescentes escolares brasileiros conforme supervisão dos pais. Pesquisa Nacional de Saúde do Escolar, Brasil, 2015.

Supervisão dos pais		Iniciação sexual	Preserv. primeira	Preserv. última	Usou MC última	Nº de parceiros (β)
Escore Supervisão (análise não ajustada)						
	0	Ref.	Ref.	Ref.	Ref.	Ref.
	1	0,70 (0,63–0,78)	1,07 (0,92–1,26)	1,22 (1,04–1,43)	1,00 (0,83–1,23)	-0,37 (-0,69;-0,06)
	2	0,57 (0,52–0,63)	1,16 (1,00–1,34)	1,24 (1,06–1,45)	1,03 (0,86–1,24)	-0,41 (-0,72;-0,09)
	3	0,44 (0,39–0,49)	1,24 (1,07–1,44)	1,35 (1,16–1,58)	1,07 (0,88–1,29)	-0,51 (-0,81;-0,21)
	4	0,37 (0,33–0,41)	1,34 (1,15–1,56)	1,49 (1,28–1,74)	1,16 (0,96–1,40)	-0,54 (-0,84;-0,25)
	5	0,34 (0,31–0,39)	1,43 (1,22–1,66)	1,57 (1,35–1,84)	1,20 (0,99–1,46)	-0,35 (-0,66;-0,04)
Escore Supervisão (análise ajustada)*						
	0	Ref.	Ref.	Ref.	Ref.	Ref.
	1	0,75 (0,68–0,84)	1,08 (0,93–1,27)	1,22 (1,04–1,43)	1,03 (0,84–1,25)	-0,40 (-0,69;-0,10)
	2	0,62 (0,56–0,69)	1,19 (1,03–1,38)	1,24 (1,06–1,45)	1,06 (0,88–1,28)	-0,50 (-0,80;-0,20)
	3	0,52 (0,47–0,58)	1,28 (1,10–1,48)	1,35 (1,16–1,59)	1,10 (0,90–1,33)	-0,59 (-0,88;-0,30)
	4	0,44 (0,39–0,48)	1,39 (1,20–1,61)	1,49 (1,28–1,74)	1,20 (0,99–1,45)	-0,66 (-0,94;-0,38)
	5	0,40 (0,36–0,45)	1,50 (1,29–1,75)	1,57 (1,34–1,84)	1,25 (1,03–1,53)	-0,54 (-0,84;-0,24)

* Valor p do teste do χ^2^ de Pearson <0,001; MC: método contraceptivo; Nº: número absoluto; Preserv.: uso de preservativo.

## DISCUSSÃO

Os achados deste estudo mostraram desigualdade de gênero no modo como os pais supervisionam seus filhos, uma vez que adolescentes do sexo masculino são menos supervisionados nas atividades voltadas para ambientes externos à casa, e as do sexo feminino, menos supervisionadas no ambiente domiciliar. No entanto, um tralho de revisão sobre o papel moderador do gênero na relação entre parentalidade e comportamento sexual de risco do adolescente sugere que o monitoramento dos pais possa ser mais protetor para adolescentes do sexo masculino com relação ao comportamento sexual de risco, enquanto para as meninas a conexão emocional com os pais pode ser um fator mais importante na prevenção de comportamentos sexuais de risco^10^. A diferença encontrada neste estudo no modo como os pais supervisionam os adolescentes de acordo com o sexo pode estar influenciada pelos modelos sociais para homens e mulheres que ainda estão presentes na sociedade, visto que, ao longo da história, as mulheres ocupavam o espaço doméstico e os homens os espaços público e político^19^. Apesar desse padrão de supervisão desigual, o efeito protetor da supervisão parental foi observado tanto em meninas quanto em meninos de forma similar (dados não mostrados).

A iniciação sexual foi mais prevalente em adolescentes do sexo masculino, e estudos prévios mostram que adolescentes do sexo masculino iniciam relações sexuais mais cedo^4,9,20,21^. Os aspectos de gênero também podem explicar essa diferença na idade da iniciação sexual, uma vez que os papéis masculinos e femininos na representação social tradicional orientam expectativas diferentes, na quais os comportamentos dos homens estão relacionados à masculinidade e virilidade, enquanto, para as mulheres, as expectativas são de que tenham maior dependência, conformismo e submissão^22^. Desse modo, o monitoramento dos pais difere de acordo com o sexo do filho^9^.

Os achados do presente estudo revelam que, entre os adolescentes que já iniciaram as relações sexuais, os comportamentos de risco à saúde sexual foram menos frequentes quanto maior o escore de supervisão dos pais. Adolescentes mais supervisionados apresentaram maior uso de preservativo na primeira e última relação sexual, maior utilização de métodos contraceptivos e menor número de parceiros sexuais. Estes resultados sugerem que a presença dos pais na vida dos adolescentes possa proporcionar condições para o compartilhamento de informações, conselhos e habilidades positivas aos adolescentes^9^, representando uma oportunidade de abordar questões referentes à sexualidade. Acrescenta-se que esse achado corrobora os de estudos internacionais^2,6–8^ e aponta o papel das práticas educativas parentais que oferecem monitoramento e aplicação de regras ao mesmo tempo que desempenham atitudes compreensivas e de proximidade como proteção contra o envolvimento dos adolescentes em comportamentos que podem conferir risco à saúde sexual, tornando-os menos suscetíveis a infecções sexualmente transmissíveis (IST) e gravidez — desfechos crescentes em nosso país entre adolescentes^23–26^.

Sobre as IST, percebe-se um aumento importante do número de casos^23,26^. Além de essas taxas terem aumentado na última década, outro fator de grande preocupação é a redução no conhecimento sobre o estado sorológico com relação ao vírus da imunodeficiência humana (HIV) e outras IST, principalmente entre as pessoas de 14 a 25 anos^23^. Esses dados, diante de nossos achados, fazem refletir sobre a necessidade constante de ações de saúde voltadas à prevenção de IST.

Outro desfecho importante neste cenário é a gravidez na adolescência, que pode levar a consequências na vida principalmente das mulheres, como a interrupção dos estudos^16^. Isso, por sua vez, impacta em longo prazo as oportunidades de completar a educação e se incorporar ao mercado de trabalho, contribuindo para situações de maior vulnerabilidade dessas mulheres e seus filhos e para a perpetuação do ciclo de pobreza e exclusão social^27^. Além disso, a gravidez na adolescência acentua as desigualdades de gênero. Incluir adolescentes homens nas ações para a redução da gravidez é desafiador, quanto mais ao se considerar a necessidade de promover reflexões sobre masculinidade, virilidade, desigualdade e violência de gênero^27^. Nesse sentido, os resultados do estudo mostram que incluir a família nas ações de saúde, no sentido de fortalecer o vínculo entre pais e filhos, pode ser uma estratégia promissora para a redução dos comportamentos sexuais de risco entre adolescentes.

Estudo longitudinal americano também demonstrou que a proximidade, o monitoramento e a comunicação dos pais durante a adolescência trouxeram repercussões positivas na saúde sexual dos filhos, além de impacto no comportamento sexual subsequente, após anos da adolescência^28^. Ressalta-se, ainda, que a supervisão dos pais também se mostrou protetora para outros comportamentos de risco entre os adolescentes, como o consumo de álcool^29^, tabaco e drogas^30^ e o sedentarismo e inatividade física^31^. Esses achados reforçam a importância do vínculo entre pais e filhos, no sentido de que práticas familiares como a supervisão e presença dos pais podem proteger os adolescentes de desfechos negativos em saúde.

Destaca-se que outros fatores também contribuem para os comportamentos sexuais e reprodutivos dos adolescentes, tais como escolaridade, uso de drogas lícitas e ilícitas, influência dos pares, religião, relações de namoro, acesso aos serviços de saúde e educação, entre outros^8,32^. Acrescenta-se que, além da família, a escola e os serviços de saúde devem desempenhar papel estratégico na formação e na educação de crianças e adolescentes, devendo ser entendidos como dispositivo central na rede de promoção à saúde de crianças e jovens^2,3^. Para isso, iniciativas intersetoriais entre educação e saúde, como o Programa Saúde na Escola (PSE), são fundamentais para a atenção integral à saúde dos escolares. Nesse âmbito, o profissional enfermeiro assume o protagonismo ao traçar ações efetivas para o público adolescente a fim de construir conhecimento e discussão, sendo capaz de contribuir para melhores práticas de saúde, em conjunto com pais e escolas, para fortalecer o vínculo e reduzir comportamentos de risco entre esses jovens^33^.

Esses programas e políticas são essenciais para se avançar no âmbito da saúde sexual e reprodutiva dos adolescentes, até bem pouco tempo uma prioridade da atenção básica no país^34^. Assim, acredita-se que esta pesquisa contribua para a construção do tema, tendo em vista o cenário incerto das políticas públicas nesse campo no país, uma vez que alguns retrocessos têm sido observados. Entre eles, destacam-se: a não assinatura do compromisso de nosso país com a Organização Mundial da Saúde (OMS) acerca da saúde sexual e reprodutiva das populações^35^; a retirada das cadernetas dos adolescentes de circulação, especialmente das informações relativas à educação sexual e sexo seguro^36^; o incentivo a políticas de abstinência sexual, que já foram consideradas ineficazes^37^; cortes orçamentários^18^; além de vários outros retrocessos previamente descritos, que podem sustentar o argumento de um possível esvaziamento programático sobre a saúde sexual e reprodutiva no país^17^.

O estudo apresenta algumas limitações, como a inclusão apenas de adolescentes que frequentavam a escola, visto que dados do IBGE^38^ apontam que 15,0% dos adolescentes com 15 a 17 anos de idade estavam fora da escola em 2015. Outra limitação deste estudo é que ele utiliza uma medida *proxy* de monitoramento parental, não sendo capaz de abordar todos os aspectos relacionados à supervisão dos pais. As diferentes chances de iniciação sexual em diferentes idades também são um limite a ser considerado, o que foi corrigido com ajuste das estimativas para essa variável.

É importante destacar que o escore somou supervisão de diferentes naturezas, como aquelas de limites/reponsabilidades e as de diálogo e afeto. No entanto, observa-se uma relação que sugere dose-resposta de que, quanto maior a supervisão, menores os comportamentos considerados de risco, o que pode significar que ambos os elementos são importantes no contexto da supervisão parental.

Destaca-se ainda que, mesmo com o lançamento dos macrodados da PeNSE 2019, a dificuldade de acesso às bases de dados dessa pesquisa sobre a saúde dos adolescentes no Brasil impede a avaliação mais atual desses indicadores e dessa relação ao longo do tempo. Por fim, acredita-se que a pandemia tenha causado grandes alterações na supervisão dos pais e na saúde sexual e reprodutiva dos adolescentes, o que merece investigações futuras.

Apesar dessas limitações, por se tratar de um inquérito com representatividade nacional que entrevistou mais de cem mil adolescentes, considera-se que os achados deste estudo são de grande relevância para o país, já que poucos trabalhos investigaram de maneira abrangente a supervisão dos pais e o comportamento sexual dos adolescentes.

Esta investigação mostrou que os adolescentes mais supervisionados pelos pais têm comportamentos sexuais mais responsáveis, mesmo que tenham sido observadas desigualdades de gênero quanto à supervisão parental. Os resultados evidenciam o papel fundamental da família em proporcionar aos adolescentes, por meio do monitoramento, simultâneo ao diálogo e ao afeto, condições estimuladoras do comportamento sexual saudável e livre de riscos.
